# Predictors of Good Clinical Outcome After Endovascular Treatment for Acute Ischemic Stroke due to Tandem Lesion in Anterior Circulation: Results from the ASCENT Study

**DOI:** 10.1007/s00270-023-03649-x

**Published:** 2024-01-12

**Authors:** Roman Havlíček, Daniel Šaňák, David Černík, Jarmila Neradová, Norbert Leško, Zuzana Gdovinová, Martin Köcher, Filip Cihlář, Jozef Malik, Jakub Fedorko, Piotr Pedowski, Jana Zapletalová

**Affiliations:** 1 Comprehensive Stroke Center, Department of Neurology, Palacký University Medical School and University Hospital Olomouc, Olomouc, Czech Republic; 2 Comprehensive Stroke Center, Department of Neurology, Central Military Hospital Prague, Prague, Czech Republic; 3 Comprehensive Stroke Center, Department of Neurology, Masaryk Hospital, KZ a.S., Ústí Nad Labem, Czech Republic; 4 Department of Neurology, P.J. Šafarik University, Faculty of Medicine and University Hospital L. Pasteur Košice, Košice, Slovakia; 5 Department of Radiology, Palacký University Medical School and University Hospital Olomouc, Olomouc, Czech Republic; 6 Department of Radiology, J. E. Purkinje University, Masaryk Hospital, KZ a.S., Ústí Nad Labem, Czech Republic; 7 Department of Radiology, Central Military Hospital Prague, Prague, Czech Republic; 8 Department of Radiodiagnostics and Imagine Techniques, P.J. Šafarik University, Faculty of Medicine and University Hospital L. Pasteur Košice, Košice, Slovakia; 9 Department of Biophysics and Statistics, Palacký University Medical School Olomouc, Olomouc, Czech Republic

**Keywords:** Acute ischemic stroke, Endovascular treatment, Tandem lesion, Clinical outcome

## Abstract

**Purpose:**

Endovascular treatment (EVT) of tandem lesion (TL) in anterior circulation (AC) acute ischemic stroke (AIS) represents still a clinical challenge. We aimed to evaluate selected factors related to EVT and assess other possible predictors of good clinical outcome besides the generally known ones.

**Methods:**

AIS patients with TL in AC treated with EVT were enrolled in the multicenter retrospective ASCENT study. A good three-month clinical outcome was scored as 0–2 points in modified Rankin Scale (mRS) and achieved recanalization using the TICI scale. Symptomatic intracerebral hemorrhage (SICH) was assessed using the SITS-MOST criteria. Logistic regression analysis was used for the assessment of possible predictors of mRS 0–2 with adjustment for potential confounders.

**Results:**

In total, 300 (68.7% males, mean age 67.3 ± 10.2 years) patients with median of admission NIHSS 17 were analyzed. Recanalization (TICI 2b-3) was achieved in 290 (96.7%) patients and 176 (58.7%) had mRS 0–2. Besides the age, admission NIHSS and SICH, admission glycemia (*p* = 0.005, OR: 0.884) the stent patency within the first 30 days after EVT (*p* = 0.0003, OR: 0.219), dual antiplatelet therapy (DAPT) started within 12 h after EVT (*p* < 0.0001, OR: 5.006) and statin therapy started within 24 h after stenting (*p* < 0.0001, OR: 5.558) were found as other predictors.

**Conclusion:**

Admission glycemia, start of DAPT within 12 h and statin therapy within 24 h after EVT, and stent patency within the first 30 days after EVT were found as other predictors of good three-month clinical outcome in AIS patients treated with EVT for TL.

**Graphical Abstract:**

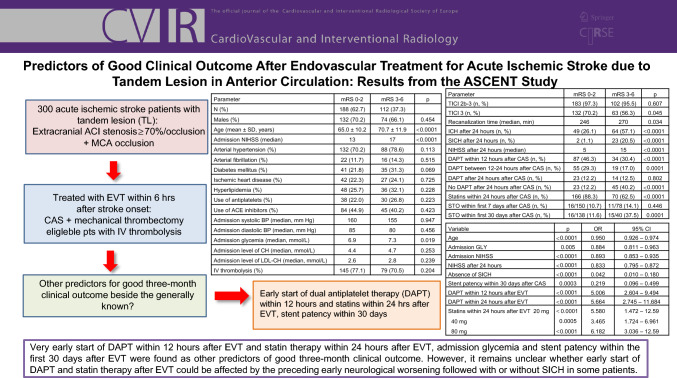

## Introduction

Emergent carotid stenting (CAS) is being performed during the endovascular mechanical thrombectomy (MT) for acute ischemic stroke (AIS) due to tandem lesion (TL) in anterior circulation (AC), however sufficient evidence from randomized trials and clear guidelines for optimal management strategy including prior IV thrombolysis (IVT) and periprocedural and post-interventional antithrombotic treatment are still missing [1–3].

Results of previous observational studies, data from patients' registries, and several meta-analyses showed mostly the safety and clinical efficacy of CAS for TL, but some of results were conflicting or controversial [4–12]. Particularly, the optimal regimen of antithrombotic therapy during and after the CAS is still being largely discussed for a possible risk of early occlusion of carotid stent and for the risk of symptomatic intracerebral hemorrhage (SICH) occurrence, especially in a case of previous IV thrombolysis [8, 13–16]. Regarding these facts, well-established regiment of antithrombotic therapy may represent one of the key points in the treatment management in such patients.

Recently, IVT was found independent predictor of good 3 month clinical outcome after endovascular treatment (EVT) for TL [15] and IVT was associated with higher odds of favorable outcome and successful intracranial recanalization [8].

The aim of our study was to evaluate selected factors and parameters related to EVT with emergent CAS, and assess other possible predictors of good clinical outcome after EVT for TL in AIS patients besides the already known ones.

## Methods

We analyzed AIS patients treated with EVT for TL in AC within the first 6 h from stroke onset, who were enrolled in the retrospective multicenter ASCENT (Acute Ischemic Stroke Due to Tandem Lesion in Anterior Circulation: Factors Influencing Technical and Clinical Results of Endovascular Treatment) study. Four participated comprehensive stroke centers provided retrospectively collected detailed technical parameters and selected clinical data from all AIS patients treated with EVT for TL between years 2016 and 2022. The obtained data from all participating centers were collected and analyzed anonymously. The study was approved by the multicenter Ethics Committee of our hospital. Individual informed consent of each enrolled patient was not required for this study analysis.

Tandem lesion was defined as a proximal intracranial occlusion (intracranial occlusion of internal carotid artery [ICA] or occlusion of M1-M2 segments of the middle cerebral artery [MCA]) and a cervical ICA occlusion or stenosis ≥ 70%. All patients underwent computed tomography (CT) of brain including CT angiography or magnetic resonance imaging (MRI) including MRI angiography.

Admission neurological deficit was assessed using the National Institutes of Health Stroke Scale (NIHSS) by a neurologist. All patients underwent brain imaging including angiography of brain. Eligible patients were treated with IVT prior EVT. Cervical carotid angioplasty with a stent placement was performed in all patients.

Mechanical thrombectomy was performed using stent-retrievers, or thrombus aspiration or combination of both. The first step treatment option in individual patient was used according to a radiologist's decision. Achieved recanalization status was assessed according to the Thrombolysis in Cerebral Infarction Scale (TICI) on the final angiogram [17].

Most patients with placed stent into extracranial ICA received intravenous acetylsalicylic acid (ASA) during EVT, and in most of those with previous IVT, the dose of intravenous ASA was reduced to 250 mg. Dual antiplatelet treatment (combination of 100 mg of acetylsalicylic acid and 75 mg of clopidogrel) was started after EVT followed with switching on a long-term single antiplatelet regimen after three months in the patients after carotid stenting. A loading dose of clopidogrel after EVT was not used in any of patients.

The occurrence of intracerebral hemorrhage (ICH) was assessed on the follow-up CT or MRI after 24 h. Symptomatic ICH (SICH) was defined as a local remote parenchymal hematoma (type 2) or subarachnoid hemorrhage associated with at least four-point increase in the NIHSS score or leading to death [18]. The stent patency after EVT was evaluated by a routine ultrasound examination during the follow-up.

Neurological deficit was evaluated using the NIHSS after 24 h and clinical outcome after three months using the modified Rankin scale (mRS). A score of 0–2 points was considered a good outcome.

### Statistical Analysis

SPSS software (version 22.0; SPSS, Chicago, Illinois) was used for statistical analysis. Fisher's exact test, chi-square test and Mann–Whitney *U* test were used for comparison of selected groups. Data normality was tested using Shapiro–Wilk test. Univariate and multivariate logistic regression analysis (LRA) was used to assess possible predictors of good clinical outcome after EVT. Multivariate model (Stepwise Forward) was adjusted for potential confounders (age, admission NIHSS, NIHSS after 24 h, recanalization time, achieved recanalization and complete recanalization [TICI 3], presence of ICH a SICH, admission glycemia, stent patency within the first seven and 30 days after EVT, decompression craniectomy for malignant brain edema, early start of DAPT and statins after EVT). All tests used an *α*-level of 0.05 for significance.

## Results

In total, 300 (68.7% males, mean age 67.3 ± 10.2 years) patients with median of admission NIHSS 17 were analyzed. Demographic and baseline clinical characteristics of all enrolled patients are shown in Table 1. Recanalization (TICI 2b-3) was achieved in 290 (96.7%) patients and 188 (62.7%) patients reached mRS 0–2 after three months. Seventeen (5.7%) patients died within the first seven days and 39 (13.0%) patients within three months after EVT.

**Table 1:** Demographic and baseline clinical characteristics of enrolled patients: comparison between the patients with good (mRS 0–2) and poor (mRS 3–6) three-month clinical outcome after endovascular treatment for tandem lesion

Parameter	mRS 0–2	mRS 3–6	*p*
*N* (%)	188 (62.7)	112 (37.3)	
Males (%)	132 (70.2)	74 (66.1)	0.454
Age (mean ± SD, years)	65.0 ± 10.2	70.7 ± 11.9	< 0.0001
Admission NIHSS (median)	13	17	< 0.0001
Arterial hypertension (%)	132 (70.2)	88 (78.6)	0.113
Arterial fibrillation (%)	22 (11.7)	16 (14.3)	0.515
Diabetes mellitus (%)	41 (21.8)	35 (31.3)	0.069
Ischemic heart disease (%)	42 (22.3)	27 (24.1)	0.725
Hyperlipidemia (%)	48 (25.7)	36 (32.1)	0.228
Use of antiplatelets (%)	38 (22.0)	30 (26.8)	0.223
Use of ACE inhibitors (%)	84 (44.9)	45 (40.2)	0.423
Admission systolic BP (median, mm Hg)	160	155	0.947
Admission diastolic BP (median, mm Hg)	85	80	0.456
Admission glycemia (median, mmol/L)	6.9	7.3	0.019
Admission level of CH (median, mmol/L)	4.4	4.7	0.253
Admission level of LDL-CH (median, mmol/L)	2.6	2.8	0.239
IV thrombolysis (%)	145 (77.1)	79 (70.5)	0.204

*ACE* angiotensin converting enzyme, *BP* blood pressure, *CH* cholesterol, *LDL* low density lipoprotein, *IV* intravenous, *mRS* modified rankin scale, *NIHSS* National Institute of Health Stroke Scale, *SD* standard deviation

Patients with good three-month clinical outcome (mRS 0–2) were younger, had lower admission NIHSS score and lower admission glycemia (Table 1). They had also higher rate of complete recanalization (TICI 3; 70.2 vs. 56.3%, *p* = 0.045) and shorter recanalization time (time interval “stroke onset-maximal achieved recanalization” [246 vs. 270 min, *p* = 0.034]) (Table 2). Patients with mRS 0–2 had less ICH and SICH on control CT and lower NIHSS score after 24 h (Table 2). An early start of DAPT within the first 12 h after EVT was more frequently present in the patients with mRS 0–2 (46.3 vs. 30.4%, *p* < 0.0001, Table 2) as well as the start of use of statins within the first 24 h after EVT (88.3 vs. 62.5%, *p* < 0.0001, Table 2). No difference was found in the rate of occluded carotid stents within the first seven days after EVT between patients with mRS 0–2 and mRS 3–6, while the rate of occluded stents within the first 30 days was higher in patients with poor outcome (Table 2).

**Table 2:** Selected parameters related to endovascular treatment and to clinical outcomes: comparison between the patients with good (mRS 0–2) and poor (mRS 3–6) 3 month clinical outcome

Parameter	mRS 0–2	mRS 3–6	*p*
TICI 2b-3 (*n*, %)	183 (97.3)	102 (95.5)	0.607
TICI 3 (*n*, %)	132 (70.2)	63 (56.3)	0.045
Recanalization time (median, min)	246	270	0.034
ICH after 24 h (*n*, %)	49 (26.1)	64 (57.1)	< 0.0001
SICH after 24 h (*n*, %)	2 (1.1)	23 (20.5)	< 0.0001
NIHSS after 24 h (median)	5	15	< 0.0001
DAPT within 12 h after CAS (*n*, %)	87 (46.3)	34 (30.4)	< 0.0001
DAPT between 12–24 h after CAS (*n*, %)	55 (29.3)	19 (17.0)	0.0001
DAPT after 24 h after CAS (*n*, %)	23 (12.2)	14 (12.5)	0.802
No DAPT after 24 h after CAS (*n*, %)	23 (12.2)	45 (40.2)	< 0.0001
Statins within 24 h after CAS (*n*, %)	166 (88.3)	70 (62.5)	< 0.0001
STO within first 7 days after CAS (*n*, %)	16/150 (10.7)	11/78 (14.1)	0.446
STO within first 30 days after CAS (*n*, %)	16/138 (11.6)	15/40 (37.5)	0.0001

*CAS* carotid stenting, *DAPT* dual antiplatelet treatment, *ICH* intracerebral hemorrhage, *NIHSS* National Institute of Health Stroke Scale, *SICH* symptomatic intracerebral hemorrhage, *STO* stent occlusion, *TICI* thrombolysis in cerebral infarction

Overall, 25 (8.3%) patients had SICH on control CT; in 5/121 (4.1%) patients with early start of DAPT within the first 12 h after CAS, in 2/74 (2.7%) patients with DAPT started within 24 h, in 1/37 (2.7%) in patients with DAPT started after 24 h and in 17/68 (25.0%) patients without DAPT treatment after 24 h (*p* = 0.002).

LRA showed, besides the generally known predictors of good outcome after EVT (age, admission NIHSS, NIHSS after 24 h and absence of SICH), admission glycemia (*p* = 0.005, OR: 0.884), stent patency within the first 30 days after CAS (*p* = 0.0003, OR: 0.219), early start of DAPT within 12 h after EVT (*p* < 0.0001, OR: 5.006) and statin therapy started within the first 24 h after EVT (*p* < 0.0001, OR: 5.558) as other predictors of good three-month clinical outcome (Table 3). The recanalization time (*p* = 0.075, OR: 0.863) was not found as predictor of mRS 0–2. Multivariate LRA found NIHSS after 24 h as only predictor for good outcome after the adjustment for potential confounders (*p* < 0.0001, OR: 0.779, 95%CI 0.770–0.865).

**Table 3:** Significant predictors of good clinical outcome after endovascular treatment for tandem lesion: logistic regression analysis

Variable	*p*	OR	95% CI
Age	< 0.0001	0.950	0.926–0.974
Admission GLY	0.005	0.884	0.811–0.963
Admission NIHSS	< 0.0001	0.893	0.853–0.935
NIHSS after 24 h	< 0.0001	0.833	0.795–0.872
Absence of ICH	< 0.0001	0.264	0.161–0.434
Absence of SICH	< 0.0001	0.042	0.010–0.180
Stent patency within 30 days after CAS	0.0003	0.219	0.096–0.499
DAPT within 12 h after EVT	< 0.0001	5.006	2.604–9.494
DAPT within 24 h after EVT	< 0.0001	5.664	2.745–11.684
Statins within 24 h after EVT			
20 mg	< 0.0001	5.580	1.472–12.59
40 mg	0.0005	3.465	1.724–6.961
80 mg	< 0.0001	6.182	3.036–12.59

*CAS* carotid stenting, *DAPT* dual antiplatelet treatment, *EVT* endovascular treatment, *GLY* glycemia, *ICH* intracerebral hemorrhage, *NIHSS* National Institute of Health Stroke Scale, *SICH* symptomatic intracerebral hemorrhage, *STO* stent occlusion, *TICI* Thrombolysis in Cerebral Infarction

## Discussion

In our study, we found the admission glycemia, early start of DAPT within the first 12 h after EVT, start of statins treatment within the first 24 h and the stent patency within the first 30 days after EVT as the other predictors of good three-month clinical outcome after EVT for TL besides the generally known ones (age, admission NIHSS and after 24 h, and SICH) (Table 3).

Our finding of lower admission glycemia in the patients with good outcome after EVT (Table 1) might correspond to the results of a recent retrospective study, which showed that lower mRS was associated with lower admission glycemia in the diabetic patients treated with reperfusion therapy incl. mechanical thrombectomy [20]. Another recent study has shown, that a high glycemic variability in AIS patients contributed to poorer outcome after EVT [21]. Furthermore, low levels of glycemia within the first 48 h after EVT may be associated with better three-month outcome [22]. Results from the recent studies showed the association between admission glycemia and functional outcome after MT in diabetic patients [25], and also in the patients with admission NIHSS ≥ 10 and MCA occlusion [26], specifically if they presented with clinical-ASPECT mismatch and achieved early recanalization (TICI 2b-3) [26].

DAPT was started more frequently within the first 12 h after EVT in the patients with good outcome in comparison with those with poor outcome in our study (Table 2). The early and intensive antiplatelet regimen after EVT with CAS is usually applied to prevent the early stent occlusion, which may be associated with a clinical worsening, or to prevent a recurrent IS, however the current evidence about impact of antiplatelet therapy on clinical outcome after EVT for TL remains weak and controversial; the recent meta-analysis, which included the data on 1658 patients treated with EVT for TL, showed only a marginal effect of DAPT after CAS on clinical outcome [5]. Nevertheless, another large recent meta-analysis showed that intraprocedural antiplatelet therapy was associated with higher rate of good functional outcome after EVT for TL [4].

Although, the patients with good clinical outcome had started DAPT very early after EVT in our study, we did not observe a difference in the rate of the early (within the first seven days after EVT) occluded stents after CAS in comparison to those with poor outcome, who had more frequently delayed start of DAPT or they did not receive DAPT after 24 h at all (Table 2). The recent results from the ETIS registry showed that aggressive antiplatelet therapy, which included oral or intravenous inhibitors of GP IIb/IIIa or P2Y12 inhibitors, was associated with increased rate of stent patency at day 1 after CAS [19]. In our study, 40.2% patients with poor three-month outcome did not receive DAPT after 24 h; mostly due to presence of SICH or ICH on control CT, and in some patients due to serious clinical worsening after EVT.

In our study, the patients with poor 3-moth clinical outcome had a higher rate (37.5%) of occluded carotid stents within the first 30 days after EVT in comparison to those with good outcome (11.6%, Table 2) and the stent patency within the first 30 day after EVT was identified as predictor of good 3 month clinical outcome (Table 3). On the other hand, the early stent patency within the first 7 days after EVT was not found to be a predictor of good clinical outcome in our study (Table 2, 3). We may assume that in some patients a patency MCA may prevent possible clinical worsening in case of early stent occlusion after EVT. Moreover, DAPT administered early after EVT might also prevent a potential distal embolization in MCA territory in case of early stent occlusion by a thrombosis.

The early start of treatment with statins after EVT (within the first 24 h) was identified as other predictor for good clinical outcome in our study (Table 3). The recent data from the RESCUE Japan Registry 2 study showed that administration of statins after admission for AIS with large vessel occlusion and EVT was associated with better clinical outcome and lower mortality after three months [23]. It might be speculated that early use of statins after CAS may help better prevent the stent occlusion after urgent CAS, which may be associated with poor outcome, however, in our study, no difference in the rate of occluded stents within the first seven days after CAS was observed between the patients with good and poor three-month clinical outcome despite the difference in the number of the patients with early start of statins use (Table 2). Furthermore, the patients with poor outcome had more occluded stents within the 30 days after CAS in our study (Table 2). We may suggest that early occlusion of stent after EVT could not be always associated with the clinical worsening if MCA remains open and with a sufficient collateral flow. Our finding of more occluded stents after the first seven days after EVT in the patients with poor outcome might be also related to other factors; e.g., the reduction, interruption or withdrawn of DAPT and use of statins due to ICH/SICH (Table 2).

We found no association between the early start of DAPT and SICH occurrence (see Results), which was in line with previous finding of no increase in SICH associated with DAPT resulting from recent large meta-analyses [14, 24], however, we cannot exclude a potential bias representing by a fact that in some of our patients, the control CT was done earlier than the start of DAPT after EVT, and thus if ICH/SICH was present on control CT, DAPT was not started at all, or antiplatelet monotherapy was used only.

Our study has some limitations. The retrospective study design and no central blinded assessment of imaging findings were main limits. Another limitation was the fact that the strategy of EVT including CAS, periprocedural antiplatelet regimen and postprocedural management were not standardized or unified in all participating centers, thus we cannot exclude definitely that some differences among centers may have impact on some of our results. No perfusion imaging was used for the selection of patients for EVT. We cannot exclude definitely if some patients were not treated due to a higher pre-stroke mRS and based on an individual decision. The fact, that some patients did not receive DAPT due to early neurological worsening and/or presence of ICH on control CT done earlier than after the first 12 h after EVT, might limit potentially the interpretation of our findings, however no association between early start of DAPT and SICH occurrence was found in our study.

## Conclusion

In our study, AIS patients, who reached good three-month clinical outcome after EVT for TL, received more frequently DAPT within the first 12 h and statin therapy within the first 24 h after EVT in comparison to those with poor outcome. Very early start of DAPT within 12 h after EVT and statin therapy within 24 h after EVT, admission glycemia and stent patency within the first 30 days after EVT were found as other predictors of good three-month clinical outcome beside the generally known ones. However, it remains unclear whether early start of DAPT and statin therapy after EVT could be affected by the preceding early neurological worsening followed with or without SICH in some patients. Thus, a further large prospective study is needed.
